# Serum Markers of Bone Turnover Following Controlled Administration of Two Medical Cannabis Products in Healthy Adults

**DOI:** 10.1089/can.2022.0181

**Published:** 2024-02-12

**Authors:** Justyna Kulpa, Graham Eglit, Melanie L. Hill, Laura MacNair, Helena Yardley, Mark A. Ware, Marcel O. Bonn-Miller, Erica N. Peters

**Affiliations:** ^1^ Canopy Growth Corporation, Smiths Falls, Canada.; ^2^ School of Medicine, University of California, San Diego, La Jolla, California, USA.

**Keywords:** cannabis, bone marker, bone resorption, bone formation, CTx, P1NP

## Abstract

**Introduction:**

Cannabidiol (CBD) has been shown to maintain bone integrity in pre-clinical models, but little is known about the effects of delta-9-tetrahydrocannabinol (THC) on bone turnover. In this study we explored the effects of two oral medical cannabis products on normal bone homeostasis through evaluation of markers of bone resorption (carboxyl-terminal collagen crosslinks, CTx) and bone formation (procollagen type 1 N-terminal propeptide, P1NP; alkaline phosphatase, ALP).

**Methods:**

This study is an analysis of secondary data from two Phase 1 double-blind, placebo-controlled trials of Spectrum Yellow (0.9 mg THC, 20 mg CBD/mL of oil) and Spectrum Red (2.5 mg THC, 0.3 mg CBD/softgel). Healthy participants (*n*=38 men, 45 women) were randomized to receive 5–20 mg THC (CBD levels varied as a function of administered product) or placebo daily (BID) for 7 days. Bone markers were assessed at baseline, upon completion of product administration (day 8), and after a 5-day washout (day 13).

**Results:**

All bone markers were significantly higher in men at baseline (*p*≤0.008). For CTx, there was a significant day×group interaction (*F*=3.23, *p*=0.04); CTx levels were significantly lower in participants treated with Spectrum Red (*b*=−164.28; 95% confidence interval [CI], −328 to −0.29; *p*=0.04) and marginally lower in participants treated with Spectrum Yellow (*b*=−157.31; 95% CI, −323 to 8.68; *p*=0.06) versus placebo on day 8. For P1NP and ALP, there were no significant differences between treatments across study days. Bone marker values outside the reference range (RR) were observed; CTx > RR (*n*=71) was predominantly (85.9%) observed in male participants, whereas P1NP > RR (*n*=100) was more evenly distributed between sexes (53.0% in men). These were not considered clinically significant and did not differ between treatment groups.

**Conclusions:**

These are the first interventional human data on the effect of cannabinoids on biomarkers of bone turnover. Short-term treatment with CBD- or THC-dominant medical cannabis products resulted in attenuation of a marker of bone resorption. Although the attenuation was not clinically significant, this finding may be indicative of protective properties of cannabinoids in bone. Further research over longer dosing durations in individuals exhibiting bone-specific conditions (e.g., osteoporosis) is warranted. ClinicalTrials.gov IDs: ACTRN12619001723178 and ACTRN12619001450101.

## Introduction

As a result of changing cannabis regulatory frameworks and social perceptions, there is growing interest in cannabis products for both medical and recreational use. Phytocannabinoid formulations generally include varying concentrations of cannabidiol (CBD) and delta-9-tetrahydrocannabinol (THC), which have the potential to produce a variety of therapeutic effects through interaction with various molecular targets. Although the anti-inflammatory, immunomodulatory, cognitive, and cardiovascular effects of cannabis have been more widely studied,^1–4^ little is known about how phytocannabinoids affect bone health.

Bone metabolism is a continual cycle of osteoclastic bone destruction and osteoblastic bone formation, which maintain bone tissue quantity and structural integrity. Both osteoclasts and osteoblasts produce the endocannabinoids anandamide (AEA) and 2-arachidonoylglycerol (2-AG) *in vitro*.^5^ Moreover, bone and cartilage cells express cannabinoid receptors CB1 and CB2,^6^ and activation of CB2 increases osteoblast proliferation^7^ while reducing osteoclast numbers.^8^ In mice, genetic knockout of CB1 or CB2 indicates that both receptors play a role in the skeleton, but the effects are sex, age, and hormonal status dependent.^5,6,9–12^ Several other receptors relevant to cannabinoid signaling have also been implicated in bone metabolism, including GPR55 and TRPV subtypes 1, 2, 4, 5, and 6.^13,14^

There is a paucity of pre-clinical data regarding the effects of THC, a CB1/CB2/GPR55 agonist,^15,16^ on bone. In one report, THC had no significant effect on fracture healing in rats.^17^ On the contrary, CBD, which acts as an antagonist of CB1/CB2/GPR55^13,15^ but an agonist at several TRPV channels,^18–20^ has been shown to maintain bone integrity in rodent and cellular models by inducing osteoblastic bone formation^17,21–23^ and/or reducing osteoclastic bone resorption.^13,23–25^

Available human data are currently limited to cross-sectional surveys wherein exact cannabis product composition and frequency/duration of use was not controlled. Data from these epidemiological studies are conflicting; whereas one study found heavy cannabis use (>5000 lifetime cannabis smoking episodes) to be both directly and indirectly (by lowering body mass index [BMI]) associated with decreased bone mineral density (BMD), high bone turnover, and an increased risk of fracture,^26^ data from the National Health and Nutrition Examination Survey (2007–2010) suggest a lack of association between any level of cannabis use and BMD.^27^ To date, no interventional human study has explored the effect of cannabinoids on indicators of bone health.

Serum bone markers have predictive validity for eventual changes in BMD,^28,29^ and changes may be measurable more immediately following treatment initiation.^30^ This study is a secondary data analysis from two Phase 1, randomized, double-blind, placebo-controlled, multiple-dose safety, pharmacokinetics and pharmacodynamics trials of commercially available oral medical cannabis products, Spectrum Yellow oil and Spectrum Red softgels.^31,32^ The purpose of this analysis was to examine the effects of these CBD- or THC-dominant products on normal bone homeostasis through evaluation of markers of bone resorption (carboxyl-terminal collagen crosslinks [CTx]) and bone formation (procollagen type 1 N-terminal propeptide [P1NP] and alkaline phosphatase [ALP]) in healthy men and women.

## Materials and Methods

### Ethics statement

All study procedures were conducted under protocols approved by the Alfred Hospital Ethics Committee (Project No. 591/19, approved November 25, 2019; Project No. 594/19; approved December 16, 2019), in accordance with the International Conference on Harmonization Good Clinical Practice guidelines, the Declaration of Helsinki, and local Australian laws and regulations. Written informed consent was obtained from each study participant before initiation of study-related procedures.

### Study design

**FIG. 1.**
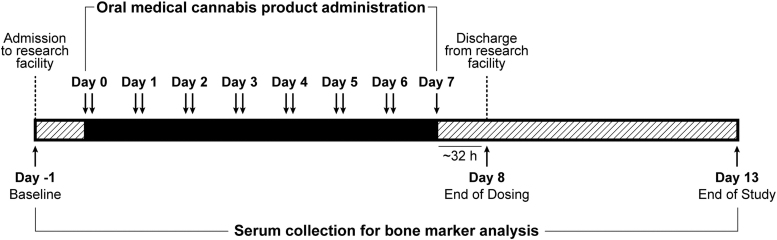
Summary of study design.

Study methodology has previously been published,^31,32^ and is summarized in Figure 1. In brief, healthy participants were recruited into each of two Phase 1, randomized, double-blind, placebo-controlled, multiple-dose trials to assess the safety, tolerability, pharmacokinetics, and pharmacodynamics of oral medical cannabis products dominant in CBD (Spectrum Yellow oil; 20 mg/mL CBD, 0.9 mg/mL THC and <0.05% terpenes in medium chain triglyceride [MCT] oil) or THC (Spectrum Red No. 2 softgels; 2.5 mg THC, 0.03 mg CBD and 0.08% terpenes in a soft gelatin capsule containing MCT, gelatin, glycerin, titanium dioxide, and color). Within each study, participants were randomized into treatment groups in which they received, in divided doses, Spectrum Yellow oil, Spectrum Red softgels, or placebo (Table 1). Study products were acquired from Canopy Growth Corporation (Smiths Falls, ON, Canada).

**Table 1.** Summary of Treatment Groups and Dose Levels Across Spectrum Yellow Oil and Spectrum Red Softgel Studies

**Product**	**CBD (mg)**	**THC (mg)**	***n* (sex)**
Spectrum Yellow	120	5.4	9 (6F:3M)
	240	10.8	8 (2F:6M)
	360	16.2	9 (4F:5M)
	480	21.6	9 (6F:3M)
Spectrum Red	0.2	5	8 (4F:4M)
	0.4	10	8 (4F:4M)
	0.6	15	8 (6F:2M)
	0.8	20	8 (5F:3M)
Placebo	0	0	16 (8F:8M)
Total			83 (45F:38M)

F, female; M, male.

Study participants were confined to a residential research facility and received study medication twice daily, approximately every 12 h after a standardized meal for 6 days, plus a single dose in the morning on day 7. Participants were discharged after a 32-h blood draw on day 8, and returned to the facility on days 9, 10, 11, and 13 for blood draws and study assessments.

### Bone markers

Blood samples for assessment of markers of bone resorption (CTx) or bone formation (P1NP, ALP) were collected upon admission to the residential research facility (baseline, day −1), at the 32-h postdose blood draw (day 8), and at study termination, at the 144-h postdose blood draw (day 13). Sample collection time ranged from 09:31 to 21:44 hours and differed significantly between timepoints (all pairwise comparisons *p*≤0.01), occurring later in the day on day 8 (16:39±3:12) versus day −1 (13:20±1:08) or day 13 (11:06±1:42) owing to the nature of the study design. However, there were no significant differences in sample collection time between treatments at baseline (all *p*s≥0.99), day 8 (all *p*s≥0.44), or day 13 (all *p*s≥0.80). With the exception of a small number of samples (*n*=4, day 8; *n*=3, day 13), serum was collected under fasted conditions.

All samples were shipped in primary collection tubes at room temperature to Australian Clinical Labs (CTx, ALP) or Alfred Health Pathology (P1NP) for analysis. Age- and sex-specific laboratory reference ranges (RR) were based on recommendations from the Australasian Association of Clinical Biochemists (AACB) Reference Intervals Harmonisation Project.^33,34^

### Statistics

Owing to sample size restrictions, dosing groups were pooled, and participants were grouped by treatment (Spectrum Yellow, Spectrum Red, or placebo). Descriptive statistics (means and standard deviations [SDs]) were calculated by treatment and sex for CTx, P1NP, and ALP at each timepoint. To inform future study planning, associations of baseline bone markers and prognostic factors (age, BMI, and sex) across groups were assessed using Pearson correlations and multiple regression models.

Linear mixed-effects models with a random intercept for participant were fit to evaluate treatment group differences across days. Fixed effects included treatment group, day (−1, 8, and 13), and the interaction of treatment group and day. Sex and age were included as additional fixed effects owing to their known association with bone turnover markers. Time of sample collection (minutes after midnight) was also included because of variation across sample collection days. The treatment×day interaction term was evaluated for statistical significance using multiple degree of freedom *F* tests based on type III sum of squares. Significant interactions were followed with simple effects and simple contrasts comparing treatments within each day. In the event of nonsignificant interactions, marginal means averaging over levels of treatment or day were calculated. Alpha level was corrected for pairwise comparisons using Tukey's procedure.

As an exploratory follow-up to significant Spectrum Red or Spectrum Yellow effects, the magnitude of treatment effects at each dose level within Spectrum Red and Spectrum Yellow products was evaluated using the same mixed-effects models described previously, which included a random intercept for subject and fixed effects of age, sex, treatment group (with placebo serving as the reference group), day, and the interaction of treatment group and day. Treatment effects were measured as the difference in the change from baseline to day 8 in treatment groups relative to placebo (i.e., [placebo day 8 − placebo baseline] − [treatment day 8 − treatment baseline]). Owing to the reduced sample size and resultant insufficient power of these contrasts, null hypothesis significance tests were not performed; instead, treatment effect estimates were accompanied by 95% confidence intervals (CIs) to reflect the precision of estimates.

Proportions of values outside normal laboratory RR were calculated by treatment group, sex, and day. Inferential statistics were not performed on these proportions owing to the loss of power after dichotomization.^35^

Analyses were conducted using R version 4.12^36^ or SPSS version 28. The R packages lme4^37^ and emmeans^38^ were used for mixed-effects modeling and simple-effect contrast calculation, respectively. Data figures were generated using GraphPad Prism version 8.4.2 for macOS.

## Results

### Participant characteristics

Among the total sample of 83 participants in this analysis, mean±SD [range] participant age was 27.6±6.4 [18–53] years, and BMI was 23.6±3.0 [18.3–29.6] kg/m^2^. Additional demographic data from these cohorts have previously been presented.^31,32^

### Evaluation of prognostic factors

Across all participants, age significantly correlated with baseline CTx (*r*=−0.40, *p*<0.01), P1NP (*r*=−0.38, *p*<0.01), and ALP (*r*=−0.22, *p*=0.04), and BMI significantly correlated with baseline CTx (*r*=−0.40, *p*<0.01) and P1NP (*r*=−0.25, *p*=0.03), but not ALP (*r*=0.06, *p*=0.61). All measured bone markers were significantly higher in male versus female participants at baseline (Supplementary Fig. S1). Collectively, age, sex, and BMI accounted for 33.6% (95% CI, 17.7% to 49.5%) of the variance in baseline CTx, 22.6% (95% CI, 7.4% to 37.8%) of the variance in baseline P1NP, and 19.8% (95% CI, 5.1% to 34.6%) of the variance in baseline ALP.

### Quantitative levels of bone markers

**FIG. 2.**
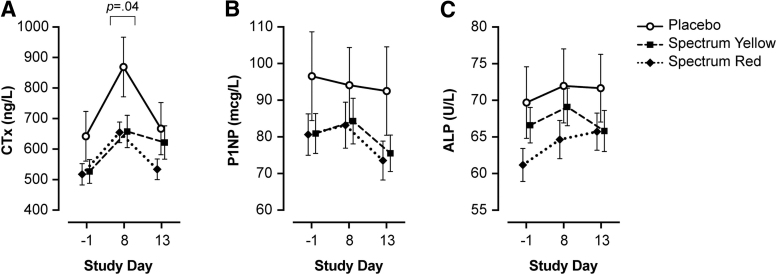
Serum bone markers following administration of placebo, Spectrum Yellow oil, or Spectrum Red softgels in healthy adults. Mean±SEM values are given for **(A)** CTx, **(B)** P1NP, and **(C)** total ALP. Blood was collected at baseline (day −1), following 7 days of twice-daily dosing (day 8), and at study termination (day 13). *p*<0.05 denotes significant difference between treatments at a given timepoint. SEM, standard error of the mean.

Results of bone marker analyses are summarized in Table 2 and Figure 2.

**Table 2.** **Mean **± **Standard Deviation [Range] Serum Bone Markers Following Administration of Spectrum Yellow Oil, Spectrum Red Softgels or Placebo in Healthy Adults**

**Treatment and sex**	**CTx (ng/L)**			**P1NP (mcg/L)**			**ALP (U/L)**		
	**Day −1**	**Day 8**	**Day 13**	**Day −1**	**Day 8**	**Day 13**	**Day −1**	**Day 8**	**Day 13**
Placebo (*n*=16)	642.0±326.1 [252–1440] (5)	868.3±392.0 [261–1857] (9)	667.2±330.5^a^ [217–1573] (6)	96.6±48.7 [45–240] (9)	94.1±39.9^a^ [42–216] (9)	92.5±46.9^a^ [42–233] (7)	69.8±19.8 [42–122]	72.1±20.4 [41–119]	71.8±18.1^a^ [46–115]
Female (*n*=8)	459.6±149.0 [252–734]	655.4±200.4 [261–891] (2)	515.4±194.8 [217–755]	78.8±23.6 [49–114] (5)	83.4±19.7^b^ [60–115] (5)	79.6±28.2 [49–134] (4)	63.6±14.2 [42–90]	65.4±13.4 [41–84]	66.6±12.1 [47–85]
Male (*n*=8)	824.4±360.1 [388–1440] (5)	1081.3±430.7 [514–1857] (7)	840.7±380.5^b^ [346–1573] (6)	114.5±61.6 [45–240] (4)	103.4±51.3 [42–216] (4)	107.1±61.1^b^ [42–233] (3)	75.9±23.5 [53–122]	78.8±24.8 [53–119]	77.7±22.7^b^ [46–115]
Spectrum Yellow (*n*=35)	527.3±231.6 [231–1199] (7)	657.8±305.5^c^ [268–1559] (12)	622.2±313.9^c^ [256–1757] (12)	80.9±32.5 [34–173] (11)	84.3±35.8^c^ [36–183] (15)	75.5±28.6^c^ [31–132] (9)	66.7±14.5 [38–92]	69.2±14.9^c^ [41–95]	65.9±16.3^c^ [10–89]
Female (*n*=18)	428.8±167.2 [231–824] (1)	516.6±185.4^d^ [268–903] (1)	441.7±148.6^d^ [256–789]	70.8±28.5 [36–124] (4)	74.0±33.5^d^ [36–142] (5)	64.6±25.4^d^ [32–103] (3)	59.6±14.4 [38–87]	62.6±16.0^d^ [41–93]	61.6±14.5^d^ [41–85]
Male (*n*=17)	631.7±248.4 [278–1199] (6)	790.7±340.3 [334–1559] (11)	781.5±337.7 [360–1757] (12)	91.6±33.9 [34–173] (7)	94.1±36.2 [39–183] (10)	85.8±28.3 [31–132] (6)	74.2±10.4 [50–92]	75.4±10.9 [53–95]	70.0±17.2 [10–89]
Spectrum Red (*n*=32)	518.1±196.6 [252–1202] (5)	654.9±190.3^e^ [295–1051] (11)	534.5±186.8^f^ [157–1132] (4)	80.6±32.2 [39–176] (14)	83.2±35.6 [35–202] (16)	73.5±29.7^f^ [30–156] (10)	61.2±12.9 [32–90]	64.7±14.9 [39–102]	65.8±14.3^f^ [36–93]
Female (*n*=19)	482.2±230.9 [252–1202] (2)	587.7±192.7 [295–1051] (3)	497.9±201.0^g^ [286–1132] (1)	74.8±31.0 [39–176] (7)	80.3±34.6 [41–202] (9)	68.7±29.0^g^ [30–156] (5)	58.3±14.0 [32–90]	61.9±17.1 [39–102]	63.7±16.1^g^ [36–93]
Male (*n*=13)	570.5±121.8 [331–783] (3)	753.2±142.3 [484–966] (8)	585.1±158.7 [157–768] (3)	88.9±33.3 [42–161] (7)	87.5±38.1 [35–178] (7)	80.2±30.5 [34–135] (5)	65.4±10.1 [50–82]	68.7±10.2 [48–84]	68.8±11.5 [48–85]

Blood was collected at baseline (day −1), following 7 days of twice-daily dosing (day 8), and at study termination (day 13). Where applicable, the number of subjects with values outside the normal reference range is shown in parentheses. Owing to sample size restrictions, statistical analysis was performed on whole treatment groups (both sexes combined); bone marker data for women and men is given for illustration purposes only.

^a^ *n*=15.

^b^ *n*=7.

^c^ *n*=33.

^d^ *n*=16.

^e^ *p*<0.05 between the indicated treatment and placebo.

^f^ *n*=31.

^g^ *n*=18.

ALP, alkaline phosphatase; CTx, carboxyl-terminal collagen crosslinks; P1NP, procollagen type 1 N-terminal propeptide.

For CTx, results of linear mixed-effects modeling revealed a significant treatment×day interaction (*χ*^2^=16.58, *p*=0.002). There was a significant simple effect of treatment on day 8 (*F*=3.23, *p*=0.04), but not at baseline or day 13 (all *p*≥0.23). Simple contrasts comparing groups on day 8 showed that CTx was significantly lower in participants treated with Spectrum Red versus placebo (*b*=−164.28; 95% CI, −328 to −0.29; *p*=0.04) and marginally lower in Spectrum Yellow versus placebo (*b*=−157.31; 95% CI, −323 to 8.68; *p*=0.06), but not Spectrum Yellow versus Spectrum Red (*b*=−6.97; 95% CI, −142.50 to 129; *p*=0.99). Six days after the final dose of study treatment (day 13), CTx returned to baseline levels in all groups; there was no significant difference between treatments on day 13 (*p*=0.23), nor between days −1 and 13 across treatments (*p*=0.09).

**FIG. 3.**
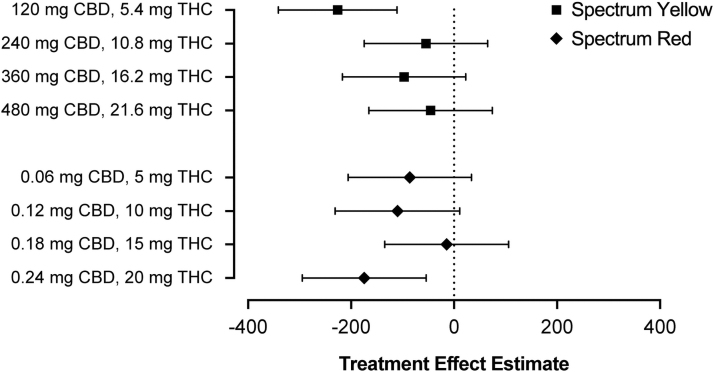
Treatment effect estimates and corresponding 95% confidence intervals for serum CTx on day 8 following treatment with Spectrum Yellow oil or Spectrum Red softgels at multiple dose levels of CBD and THC. CTx, carboxyl-terminal collagen crosslinks.

Exploratory follow-up analyses evaluating the magnitude of treatment effects at each dose level within Spectrum Yellow and Spectrum Red products revealed that treatment effect estimates were largely comparable across doses (Fig. 3). The lowest Spectrum Yellow dose (120 mg CBD +5.4 mg THC) exhibited a somewhat larger effect; however, given the small sample size for these comparisons, this may reflect sampling error. Of note, there was no appreciable difference across Spectrum Yellow and Spectrum Red products at comparable levels of THC.

For P1NP, linear mixed-effects modeling revealed that the treatment×day interaction was not significant (*χ*^2^=7.20, *p*=0.13); main effects of day and treatment were evaluated separately. Collapsing across treatments, P1NP differed significantly between baseline and day 13 (*b*=6.34; 95% CI, 2.14 to 10.59; *p*=0.001) and days 8 and 13 (*b*=6.02; 95% CI, 0.003 to 12.04; *p*=0.05), but not between baseline and day 8 (*p*=0.99). There were no significant differences between treatments (all *p*≥0.68) across study days.

For ALP, linear mixed-effects modeling revealed that the treatment×day interaction was not significant (*χ*^2^=8.44, *p*=0.08); main effects of treatment and day were evaluated separately. Collapsing across treatments, ALP differed significantly between baseline and day 8 (*b*=−3.64; 95% CI, −6.64 to −0.65; *p*=0.01), but not between baseline and day 13 nor day 8 and day 13 (*p*s>.11). There were no significant differences between treatments (all *p*s≥0.33) across study days.

Sensitivity analyses were conducted excluding participants whose blood was sampled under nonfasting conditions and all effect estimates reported above remained largely similar.

### Proportions of bone marker values outside of RR

Although several participants had bone marker values above RR for their age and sex,^33^ these were not considered clinically significant (Table 2). The number of participants with CTx or P1NP values >RR increased when comparing day −1 (CTx: *n*=17; P1NP: *n*=34) and day 8 (CTx: *n*=32; P1NP: *n*=40), decreasing by day 13 (CTx: *n*=22; P1NP: *n*=26). CTx elevation occurred disproportionately across sexes; 85.9% (*n*=61 of 71 total observations) of total CTx results >RR were observed in men. P1NP elevation was more evenly distributed between sexes; 53.0% (*n*=53 of 100 total observations) of P1NP results >RR were observed in men. ALP values remained within RR for all participants at all timepoints.

## Discussion

To date, human work assessing the effects of phytocannabinoids on indicators of bone health has been limited to epidemiological data,^26,27^ and only one previous human study explored bone turnover markers associated with cannabis use. In a cross-sectional study, both CTx and P1NP were elevated in heavy cannabis users as compared with a cigarette-smoking control group.^26^ As exact cannabis product composition and frequency/duration of use could not be controlled, the association of specific phytocannabinoids or cannabis products with bone turnover markers has not yet been established.

In our study, participants were randomized to receive one of four doses of Spectrum Yellow oil (120–480 mg CBD and 5.4–21.6 mg THC daily) or Spectrum Red softgels (0.06–0.24 mg CBD and 5–20 mg THC daily), two well characterized commercially available cannabis products. The studied dosages of THC and CBD were based on the primary aim of the parent trials to evaluate the safety, pharmacokinetics, and pharmacodynamics of THC and CBD. This large dose range was intended to closely approximate real-world conditions in individuals who consume cannabis for medical purposes and to best inform physician and patient decision-making regarding a variety of dosing parameters. Future studies with lower dosages of THC and CBD will be more relevant for individuals who consume cannabis for nonmedical purposes. Owing to sample size restrictions, we were not able to compare CBD and/or THC dose level within treatments with bone marker changes. That both Spectrum Yellow and Red products were found to produce an equal attenuation of CTx may be an indication that both CBD and THC act on this biomarker but may also be confounded by the presence of both cannabinoids, although at different concentrations, in both study products.

Sex differences in cannabinoid function, pharmacology, and receptor distribution have been noted in pre-clinical literature,^39–41^ and bone marker expression and bone disease risk are known to differ between men and women across different age groups.^42–48^ This study recruited both male and female participants but was not designed to examine differences in bone marker response between sexes. In agreement with previous literature,^42,43^ we observed higher baseline levels of all three bone markers in this healthy population. However, the overall response to cannabinoid products followed a similar pattern in men and women. Whether this uniform effect across sexes is maintained in patient populations exhibiting bone-specific conditions is unknown.

Bone markers, particularly markers of resorption, are known to follow a diurnal rhythm.^49,50^ Peak levels of serum CTx have been shown to occur in the early morning (05:00–08:00 hours), with nadir in the early afternoon (14:00 hours), rising again by late afternoon (17:00 hours). Across treatments, we observed higher CTx on day 8 (mean collection time=16:39 hours) as compared with days −1 (13:20 hours) and 13 (11:06 hours). Collection time variation may also explain the prevalence of findings outside RR, because RR is established following optimal (morning, fasted) serum collection procedures. As bone markers were an exploratory endpoint in the original study design, serum collection times were not optimized for CTx measurement.

Future work in this area should ensure serum collection is standardized across participants, occurring in the morning following an overnight fast, as fasting significantly reduces individual diurnal variations.^51^ Of importance, in this study, time of serum collection was uniform between treatments at each timepoint. Differences in CTx between participants receiving cannabis products versus placebo on day 8 are therefore expected to be owing to the products themselves, rather than serum collection timing. Bone turnover markers are also known to be affected by age, BMI, recent fracture, tobacco/alcohol intake, and in women, phase of the menstrual cycle, pregnancy, lactation, and menopausal status.^52–58^

Within person variation in the Australian population is estimated at 8% (ALP and P1NP) to 10% (CTx), whereas clinically relevant least significant change (LSC) in CTx and P1NP during antiresorptive treatment is generally defined as 2.8 times the biological variation (∼30% and ∼21%, respectively).^59^ Although not an ideal comparison, we observed a difference of −24.2% (Spectrum Yellow) and −24.6% (Spectrum Red) when oral medical cannabis products were compared with placebo treatment over a 7-day dosing period. This finding is in line with pre-clinical work, which has demonstrated ∼18% decrease in CTx following repeated CBD dosing (10 mg/kg) in healthy male mice over 8 weeks.^13^ Whether longer dosing duration and/or treatment of populations with elevated bone turnover would further attenuate CTx levels or modulate bone formation markers above LSC remains to be established.

Total ALP has been shown to be elevated in rodents and dogs,^60–63^ but uncommonly in humans,^64,65^ following repeated administration of cannabinoids, particularly CBD. In agreement with existing human data, cannabinoid administration did not result in ALP elevation in this study. Although ALP activity is often associated with liver function, serum ALP also originates from several other tissues, including bone (∼50% of total ALP).^30^ Total ALP activity has traditionally been used as a therapeutic marker in multiple bone conditions,^66^ but tissue-specific isoform assays have become available. Future work should evaluate activity of the bone-specific ALP isoform, which is more directly correlated with the number and differentiation state of osteoblasts.^67^

This is the first interventional human study to explore the effect of cannabinoids on biomarkers of bone turnover. In healthy adults, short-term treatment with commercially available medical cannabis products, Spectrum Yellow oil and Spectrum Red softgels, did not result in clinically significant changes in bone turnover markers. However, as compared with placebo treatment, oral medical cannabis products attenuated the increase in a resorption marker, CTx, following 7 days of twice daily administration. This finding may be indicative of protective properties of cannabinoids in bone. Further research following longer term treatment in individuals exhibiting bone-specific risk factors and/or medical conditions (e.g., osteopenia, osteoporosis) is warranted.

## Supplementary Material

Supplemental data

## Abbreviations Used

ALP: alkaline phosphatase
BMD: bone mineral density
BMI: body mass index
CBD: cannabidiol
CI: confidence interval
CTx: carboxyl-terminal collagen crosslinks
LSC: least significant change
MCT: medium chain triglyceride
P1NP: procollagen type 1 N-terminal propeptide
RR: reference range
SD: standard deviation
SEM: standard error of the mean
THC: delta-9-tetrahydrocannabinol
