# Supplementary material for: Serum Markers of Bone Turnover Following Controlled Administration of Two Medical Cannabis Products in Healthy Adults
Source: Cannabis Cannabinoid Res. 2024 Feb 12;9(1):300–9. doi: 10.1089/can.2022.0181 (PMC10874824; doi:10.1089/can.2022.0181)

**SUPPLEMENTARY MATERIALS**

**Supplementary Figure 1.** Sex differences in serum bone markers at baseline in healthy adults. Mean ± SD are shown for A) carboxyl-terminal collagen crosslinks (CTx), B) procollagen type 1 N-terminal propeptide (P1NP), and C) alkaline phosphatase (ALP) across female and male study participants. *p*<.05 denotes significant difference between sexes.


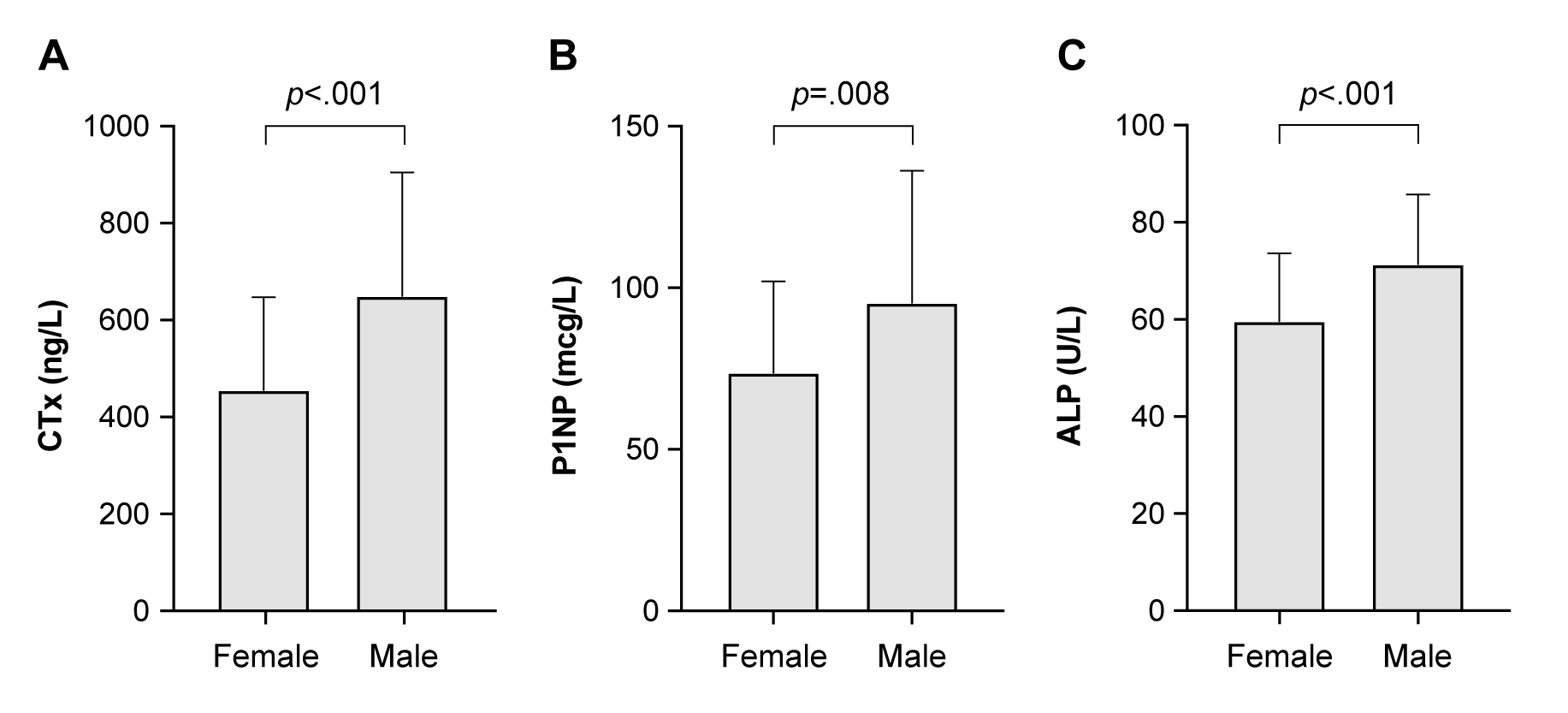

Supplement: Supplemental data [file Suppl_FigS1.docx]
